# Spef1/CLAMP binds microtubules and actin‐based structures and regulates cell migration and epithelia cell polarity

**DOI:** 10.1111/nyas.14845

**Published:** 2022-06-16

**Authors:** Rocio Tapia, Gail A. Hecht

**Affiliations:** ^1^ Division of Gastroenterology and Nutrition, Department of Medicine Loyola University Chicago, Loyola University Medical Center Maywood Illinois USA; ^2^ Department of Microbiology and Immunology Loyola University Chicago, Loyola University Medical Center Maywood Illinois USA

**Keywords:** actin, intestinal epithelial cells, microtubules, multiciliated cells, planar cell polarity, tight junctions

## Abstract

During migration, cells invade, repair, and create barriers leading to the formation of new cellular contacts in target tissues. Cell migration requires many proteins that collectively form the cytoskeleton. The main cytoskeletal elements are actin filaments, microtubules (MTs), and intermediate filaments. These structures work in concert with a large number of accessory proteins that contribute in a variety of ways to regulate filament assembly and turnover, to alter the configuration or arrangement of filaments by bundling or crosslinking, to link the cytoskeleton to other structures in the cell, such as membranes and junctions, and to transport cargo along the filaments. Sperm flagella protein‐1 (Spef1), also designated calponin homology and microtubules‐associated protein (CLAMP), is a multifunctional protein that interacts with cytoskeletal structures, including MTs, actin filaments, and focal adhesions in epithelia. In this review, we outline Spef1/CLAMP structure and expression in several cellular models. The function of Spef1/CLAMP in flagellar and ciliary motility, MT‐binding and stability, regulation of planar cell polarity, and potential contribution to the maintenance of actin‐based structures, such as lamellipodia and filopodia during cell migration, are also discussed.

## Graphical abstract

Sperm flagella protein‐1 (Spef1), also designated calponin homology and microtubules associated protein (CLAMP), is a multifunctional protein that interacts with cytoskeletal structures including microtubules, actin filaments, and focal adhesions in epithelia. In this review, we outline Spef1/CLAMP structure and expression in several cellular models.

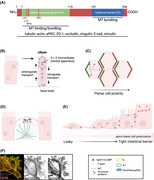

## BRIEF DESCRIPTION OF SPEF1/CLAMP

In 2005, Chan *et al*. first identified and characterized a novel mouse gene, *Spef1*, that is expressed in testis and is present in the flagellum of the mature spermatozoon.^1^ *Spef1* has evolutionary orthologs in a wide range of species, including mammals, other vertebrates with motile cilia or flagella, *Drosophila*, chordates, such as *Ciona intestinalis*, platyhelminthes, and protozoans including *Chlamyodomonas*, *Plasmodium*, *Giardia, Leishmania*, and *Trypanosoma*.^2^, ^3^, ^4^, ^5^, ^6^, ^7^ Multiple sequence alignment of *Spef1* orthologs shows that there are two distinct members of the Spef protein family, Spef1 and Spef2. In this review, we mainly focus on and discuss the physiological role of Spef1.

In multicellular organisms, both *Spef1* and *Spef2* have been detected in expressed sequence tag libraries from testis or spermatocytes. *Spef1* mRNA expression is mainly detected in the testis, and very faint levels are detected in the epididymis, vas deferens, and sperm. In the testis, *Spef1* mRNA is not present prior to 3 weeks or in the early stage of embryonic development.^1^ Importantly, *Spef1* mRNA is upregulated during multiciliated differentiation of mouse tracheal epithelial cells (mTECs).^8^ The importance of *Spef1* to cell development and cell survival has been demonstrated in *Xenopus leavis* embryos and intestinal epithelia cells, respectively. Werner *et al*. found that high doses of a Spef1 morpholino were lethal to *Xenopus* embryos around stage 14, before multiciliated differentiation. Additionally, CRISPR‐mediated genome editing results in a complete loss of cell viability, suggesting a broad developmental role for *Spef1*.^9^, ^10^

Spef1, also designated CaLponin homology and Microtubules‐Associated Protein (CLAMP), is localized proximal to the lumen of seminiferous tubules and is abundant in testis, sperm extracts, lung, oviduct, trachea, pillar cells of the organ of Corti, and human colonic tissues.^1^, ^8^, ^10^, ^11^ In cultured polarized cells, Spef1/CLAMP is present in human intestinal enteroids, colonic intestinal cells (SKCO‐15), Madin–Darby canine kidney cells, mosaic tissues of *Xenopus* embryos, and mTECs and mouse multiciliated ependymal cells (mEPCs).^8^, ^9^, ^10^, ^12^ Although Spef1/CLAMP mRNA and protein have been detected in several tissues, its function is not fully understood. In this review, we will integrate all the studies reported of Spef1/CLAMP and we will discuss its potential functions in several tissues, where this protein has been localized.

**FIGURE 1:**
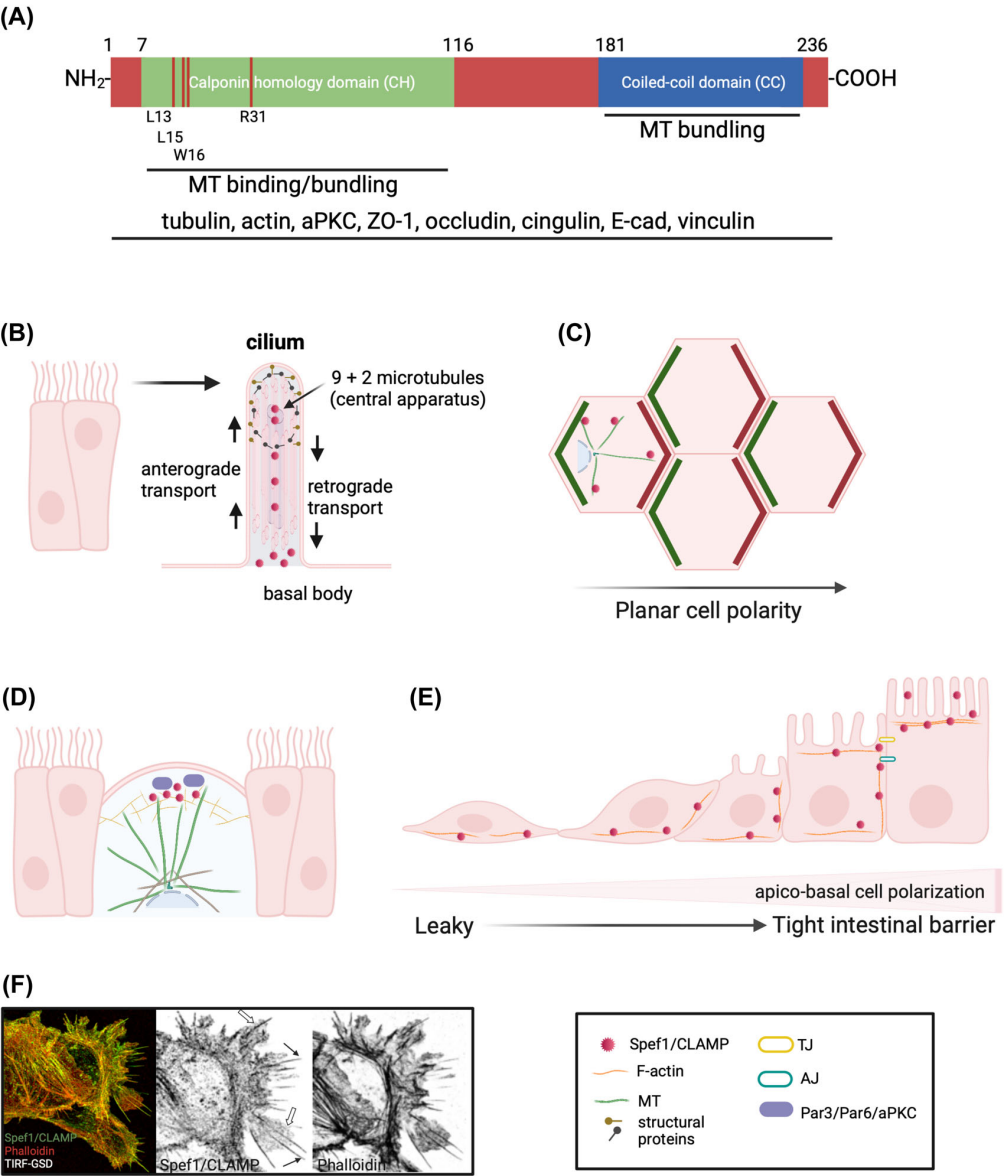
Spef1/CLAMP in epithelial cells. (A) Cartoon depicting the Spef1/CLAMP protein structure. The N‐terminal region of Spef1/CLAMP contains a calponin homology (CH) domain and its carboxy terminus has a coiled‐coil (CC) domain. The CH domain of Spef1/CLAMP contains crucial amino acids for binding and bundling MTs (R31), and conserved putative actin binding surface residues (L13, L15, and W16). Spef1/CLAMP binds MTs through its CH domain, while both the CH and CC domains are required for bundling MTs. In epithelial cells, Spef1/CLAMP forms a complex with several proteins, such as tubulin, actin, aPKC, ZO‐1, occludin, cingulin, E‐cadherin, and vinculin. (B–D) Spef1/CLAMP localization and function in multiciliated cells. (B) Spef1/CLAMP is localized at the ciliary rootlet (basal body) along the axoneme and apical tips of cilia, colocalizing with microtubules (MTs) and MT‐associated proteins. (C) Spef1/CLAMP regulates PCP signaling via asymmetric localization of cell polarity components and MTs. (D) Spef1/CLAMP associates with Par polarity proteins to stabilize MTs at the apical membrane of multiciliated migrating cells. (E, F) Spef1/CLAMP localization and function in intestinal epithelial cells. (E) Spef1/CLAMP is differentially expressed and localized at the basal membrane of undifferentiated cells (leaky intestinal barrier). At the lateral membrane, Spef1/CLAMP interacts with adhesion complexes (TJs and AJs). In fully polarized epithelia (tight intestinal barrier), Spef1/CLAMP accumulates at the apical membrane in microvilli. (F) Spef1/CLAMP is enriched in lamellipodia (white arrows) and filopodia (black arrows) of migrating intestinal epithelial cells. Created with BioRender.com

Spef1/CLAMP encodes a predicted protein of 236 amino acid residues with a molecular weight of ∼27 kDa and contains two distinct domains of unknown function (Figure 1A). Spef1/CLAMP contains a highly conserved amino‐terminal region that harbors a calponin‐homology domain (CH) (residues 7–116) and a coiled‐coil (CC) domain in the carboxy terminus (residues 181–234). In addition, Spef1/CLAMP harbors potential phosphorylation and glycosylation sites, a nuclear localization signal, MT‐binding and ‐bundling sites, and potential actin‐binding residues within its protein sequence.^1^, ^8^, ^10^, ^11^

## SPEF1/CLAMP BINDS/BUNDLES MT, CONTRIBUTES TO MT STABILITY, AND REGULATES THE MOTILITY AND ROTATIONAL MOVEMENT OF FLAGELLA AND CILIA

Spef1/CLAMP has also been identified as a conserved component of *T. brucei* flagella (TbSpef1). *T. brucei* is the causative agent of African trypanosomiasis, also known as sleeping sickness, in humans. *T. brucei* has a single flagellum, which is essential for viability and motility and has emerged as a key player in multiple facets of development, transmission, and pathogenesis.^13^ TbSpef1 binds an MT quartet (MTQ) in the flagella, which originates from the basal bodies and extends toward the anterior end of the cell. MTQ‐basal body anchorage is critical for the spatial organization of cytoskeletal organelles, as well as the morphology of the membrane‐bound flagellar pocket of *T. brucei*. TbSAF1 is a basal body protein required for anchoring the TbSpef1–MTQ complex to basal bodies of the flagella. Depletion of TbSpef1 causes severe motility defects, whereas depletion of TbSAF1 leads to dissociation of the TbSpef1–MTQ complex from the basal bodies, leading to changes in the morphology of the flagellar pocket of *T. brucei*.^7^, ^14^

The sperm flagellum is organized into three structural regions: the middle, principal, and end piece. The internal structure of the flagellum is similar to that of flagella and cilia found in other cell types and organisms. The motile cilium is typically comprised of a 9 + 2 MT organization, nine doublet MTs surrounding of a pair of central MTs (Figure 1B). The central MTs form a scaffold for the assembly of protein complexes forming a network of interconnected projections. The central axoneme of MTs and associated structures are collectively referred to as the central pair apparatus (CP).^15^ This axonemal structure contributes to flagellar and ciliary motility. In the sperm flagellum, the axoneme is surrounded by structures termed the outer dense fibers (ODFs) and the fibrous sheath (FS).^16^ The FS and ODFs play an important role in flagellar motility by controlling the flexibility of flagella in a mechanical manner. In the flagellum, Spef1/CLAMP is present between ODFs and within the FS. Interestingly, it has been found that as spermatids mature, Spef1/CLAMP expression is strongest and most luminal within the cytoplasm of the spermatids. This pattern of localization may correspond to the synthesis of the protein during spermatogenesis and its subsequent incorporation into flagella of maturing spermatids prior to spermiation.^1^ Although it is not clear whether Spef1/CLAMP associates with ODF and FS structures, its localization might indicate that Spef1/CLAMP is an important component of the mature sperm tail and might be involved in spermatogenesis.

Spef2 is a cilia‐associated protein highly expressed in various ciliated tissues, such as lung, spleen, trachea, brain, and testis.^17^ It has been reported that Spef2 plays an important role in sperm tail development and sperm motility. Spef2 interacts with the intraflagellar transport 20 protein, which is essential for male fertility and spermiogenesis.^18^ Defects in the splicing of an isoform of Spef2 or dysfunctional mutations in this gene impair sperm motility and cause a short‐tail phenotype in pig and mice models.^19^, ^20^ Male patients with multiple morphological abnormalities of the sperm flagella show mutations in *Spef2*, resulting in disrupted axonemal structure, reduced sperm motility, and sperm tail defects, suggesting loss‐of‐function mutations in *Spef2*.^21^, ^22^ Although there are no data regarding the function of Spef1/CLAMP at the sperm tail, it can be suggested that this protein, through its associations with several sperm tail components, might participate in the maintenance of the structure and motility of the sperm flagella in a fashion similar to Spef2.

In *Xenopus* embryos, exogenous Spef1/CLAMP localizes at the ciliary rootlet and axonemes and is enriched at the apical tips of cilia.^23^, ^24^ Fuz, a planar cell polarity (PCP) effector, is essential for ciliogenesis in *Xenopus*, participates in the trafficking of cargo molecules to basal bodies and to the apical tips of cilia, and is critical for mouse embryonic development.^23^, ^24^ Fuz interacts with the Rab‐related small GTPase RSG1 and is essential for ciliogenesis and secretion. The Fuz/RSG1 complex contributes to Spef1/CLAMP localization and possibly alters its function in cilia.^24^ In mosaic epidermis of *Xenopus*, Fuz morphants display shortened and dysmorphic cilia and Spef1/CLAMP is entirely absent at the apical tips. Spef1/CLAMP also localizes to the apical surface, where it forms a well‐defined linear structure adjacent to apically docked basal bodies. In Fuz morphant embryos, Spef1/CLAMP is present in larger, irregularly shaped foci positioned below the apical surface of cilia. Additionally, RSG1 morphant cells show defects in the trafficking of Spef1/CLAMP to the apical cell surface and its accumulation in the apical tips of cilia in multiciliated cells, suggesting that the Fuz/RSG1 complex is required for the apical accumulation of Spef1/CLAMP in cilia.^24^

Spef1/CLAMP is highly expressed in mTECs after being cultured at an air–liquid interface and coincident with multicilia formation. During mEPC differentiation by serum deprivation, Spef1/CLAMP is expressed in the mEPCs, and weakly or undetectable in nonciliated cycling cells, suggesting that Spef1/CLAMP expression is tightly correlated with the presence of motile cilia.^8^ During multiciliogenesis of mEPCs, Spef1/CLAMP is only observed in growing cilia and is enriched at the ciliary tip, potentially correlating with the assembly of MTs at the CP of cilia.^8^ Spef1/CLAMP is present at the central region of the 9 + 2 ependymal cilia, where it colocalizes with Hydin, an MT‐associated protein that is part of the CP complex 2 projection (Figure 1B). In multiciliated cells, motile cilia beat in a polarized and synchronized fashion to drive directed fluid flow across the epithelium. Recent evidence indicates that Spef1/CLAMP is required for the planar beating of cilia. Spef1/CLAMP deficiency in multiciliated cells of *Xenopus* and mEPCs of mouse alters the back‐and‐forth stroke pattern of cilia rotation, causing significant loss of polarity and random orientation of cilia within cells.^8^, ^12^ The loss of cilia orientation has been associated with defects in PCP signaling, suggesting that Spef1/CLAMP may play an important role in the establishment of cilia polarity.^12^ Although the mechanism underlying the role of Spef1/CLAMP deficiency in cilia motility and orientation remains unknown, the importance of motile cilia in epithelial cells of the trachea, oviduct, and ependyma, where Spef1/CLAMP is abundant, is crucial in mucus clearance, ovum transport, and cerebrospinal fluid circulation, respectively. Dysfunction of human multiciliated cells is associated with diseases of the brain, airway, and reproductive tracts.

Primary ciliary dyskinesia (PCD) is a genetically heterogeneous chronic destructive airway disease characterized by defects in motile cilia function. PCD patients commonly present chronic sinusitis, bronchiectasis, neonatal respiratory distress, male infertility, and occasionally hydrocephalus, otitis media, female infertility, and retinitis pigmentosa.^25^, ^26^, ^27^, ^28^, ^29^, ^30^, ^31^, ^32^, ^33^ Several genes have been implicated in PCD, many of which encode ciliary components, including Hydin,^34^, ^35^, ^36^ a protein that colocalizes with Spef1/CLAMP at the ciliary tips.^8^ In Hydin mutant respiratory cilia from patients with PCD and CP defects, Spef2 is absent in these mutant cells, suggesting its dependence on functional Hydin.^34^ Genetic analysis of patients with suspected PCD reveals loss‐of‐function mutations in *Spef2*, indicating that SPE has a crucial role in PCD and CP defects.^34^ Importantly, big giant head (*bgh*) mouse mutants have several abnormalities associated with PCD, including hydrocephalus, male infertility, and sinusitis. *bgh* mutant mice have short flagella and disorganized axonemal structures in elongating spermatids and mature sperm. The beat frequency of *bgh* mutant respiratory cilia is lower than that of wild‐type cilia.^20^ Mutation of *Spef2* results in the PCD phenotype in *bgh* mutant mice due to a reduction in respiratory ciliary beating.^20^ Although the mechanisms involved in the cilia and flagella defects caused by Spef1/CLAMP are still unknown, collectively, these data support the notion that Spef1/CLAMP has multiple tissue‐specific functions. In epithelial multiciliated cells, Spef1/CLAMP may regulate ciliary motility, whereas in testis, it may be important for sperm biogenesis, functions similar to those of Spef2.

Dougherty *et al*. found that Spef1/CLAMP localizes throughout extended MT bundles of mature pillar cells in the cochlea.^11^ In epithelial cells (e.g., Cos‐7, SKCO‐15, RPE‐2, U2OS, and HEK293T), ectopic expression of Spef1/CLAMP associates with and bundles MTs, and protects MTs against cold/nocodazole‐induced MT depolymerization, suggesting that Spef1/CLAMP contributes to MT stabilization.^8^, ^9^, ^10^, ^11^ It has been proposed that Spef1/CLAMP associates with MTs via its CH domain and may function as an MT‐associated protein. The amino terminus of Spef1/CLAMP shares ∼22% identity to EB3 and 19% identity to EB1 in their respective CH domain regions.^11^ EB proteins mediate MT bundling through their CH domains. Importantly, Spef1/CLAMP harbors a conserved arginine residue at position 31 (Arg31) within its CH domain, which is crucial for both MT‐binding and ‐bundling activities. Expression of MT‐binding‐defective mutant (Spef1/CLAMP^R31A^) disrupts both MT‐binding and ‐bundling activities in cells. CC domain deletion mutants of Spef1/CLAMP (Spef1/CLAMP^ΔCC^) bound MT but failed to bundle them. Therefore, the CH domain of Spef1/CLAMP appears to be responsible for MT‐binding activity, while both the CH and CC domains are required for bundling MT. Furthermore, cilia of ependymal cells deficient in Spef1/CLAMP or expressing Spef1/CLAMP^R31A^ mutants lack central MTs and are unable to rescue CP MTs. Additionally, Spef1/CLAMP^R31A^ and Spef1/CLAMP^ΔCC^ mutants fail to rescue ciliary beat defects and ciliary retention of the CP proteins Spag6 and Hydin.^8^ These data suggest that Spef1/CLAMP, through its conserved MT‐associated role and interaction with MT‐associated proteins, such as Hydin and Spag6, is essential for CP formation and maintenance of cilia function.

## SPEF1/CLAMP BINDS ACTIN

It has been proposed that two different pools of Spef1/CLAMP exist within epithelia, each having distinct localization and function within cells. Kim and colleagues found that endogenous Spef1/CLAMP weakly localizes to the MT network and is enriched at sites of increased MT concentration, such as a centrioles and rootlets associated with motile cilia.^12^ Importantly, significant accumulation of endogenous Spef1/CLAMP is observed at apical cell–cell junctions.^10^, ^12^ Although the role of Spef1/CLAMP at cell–cell contacts remains unknown, it has been shown that Spef1/CLAMP binds actin filaments and regulates actin dynamic structures.^10^

Recently, our group demonstrated that Spef1/CLAMP shares high homology with several cytoskeletal proteins that bind MT and actin.^10^ The full‐length sequence of Spef1/CLAMP shares homology with cytoskeletal proteins, such as ACF7, α‐parvin, nesprin, β‐spectrin, plectin, MICAL, smoothelin, utrophin, EHBP1, α‐actinin, filamin, dystrophin, and dystonin, suggesting that Spef1/CLAMP may be associated with actin‐ and MT‐based structures. Additionally, Spef1/CLAMP has a type 2 CH domain and harbors conserved amino acids (L_13_, L_15_, W_16_, N_28_, D_35_, G_36_, I_43_, P_48_, L_80_, L_104_, and L_107_) present in the CH region of many cytoskeletal proteins. Interestingly, residues L_13_, L_15_, and W_16_ within the CH domain of Spef1/CLAMP are conserved in the actin‐binding site 3 of several cytoskeletal proteins. Residue W_16_ has been reported to regulate actin binding and the spatial structure of the CH domains of filamin, spectrin, and fimbrin.^35^, ^36^, ^37^ CH domain architecture seems to be important for protein–protein interactions and functionality. Alignment and modeling data indicate that the CH domain of Spef1/CLAMP, which consists of six α‐helices, folds in a similar manner to the CH domain of EB1 and α‐actinin.^10^, ^11^

The interaction between Spef1/CLAMP and actin has been demonstrated using several approaches. The proximity ligation assay, which indicates the closeness of two proteins (<40 nm), demonstrated that Spef1/CLAMP could form a complex with actin. Super resolution microscopy (total internal reflection fluorescence‐ground state depletion microscopy), which detects molecules in proximity as close as 20 nm, shows that Spef1/CLAMP aligns along individual actin filaments, as well as at the cell edge and within lamellipodia of sparsely plated intestinal epithelial cells (Figure 1F).^10^ The close association of Spef1/CLAMP with actin was also demonstrated in intestinal epithelia infected with enteropathogenic *Escherichia coli* (EPEC), which intimately attaches to and effaces the microvilli of host cells and reorganizes the actin cytoskeleton to form actin‐rich pedestals. During EPEC infection, Spef1/CLAMP is recruited to actin pedestals underlying attached EPEC.^10^ Together, these data confirm the close association of Spef1/CLAMP with actin and suggest this complex is involved in intestinal epithelial cytoskeletal dynamics. Biochemically, Spef1/CLAMP association with actin has been demonstrated by immunoprecipitation in cell lysate extracts of intestinal epithelia.^10^ Unexpectedly, testis extracts from mice do not show Spef1/CLAMP association within the actin fraction.^1^ These controversial results suggest that Spef1/CLAMP may contribute to epithelial cytoskeletal dynamics in a cell‐specific manner and that this protein may use its CH conformation to bind preferentially distinct cytoskeletal structures. Further studies are needed to determine the biological functions of the actin and MT‐binding regions of Spef1/CLAMP.

## SPEF1/CLAMP REGULATES CELL POLARITY AND CONTRIBUTES TO CELL MIGRATION

Epithelia require apical–basal membrane polarity to perform crucial vectorial transport functions and cytoplasmic polarity to generate and facilitate tissue morphogenesis. Polarity formation is organized and executed in response to extracellular signaling and through establishment of an apical–basal axis, intercellular junctions, cytoskeletal rearrangements, and polarized trafficking machinery. Two types of cell polarity, apicobasal polarity and PCP, have been described in epithelia. Apicobasal polarity contributes to cell morphology, directional vesicle transport, ion and solute transport, and specific localization of proteins and lipids to different membrane domains.^38^ PCP is an essential feature of animal tissues and is established within the plane of a cell sheet. Polarization propagates throughout the whole tissue, providing a polarity axis that governs collective morphogenetic events, such as the orientation of subcellular structures and cell rearrangement.^39^, ^40^

Tight junctions (TJs) localized at the most apical region of the lateral membrane constitute a paracellular diffusion barrier modulating the flow of ions and solutes. TJs also form a fence contributing to the maintenance of apicobasal polarity by restricting the intermixing of apical and lateral plasma membrane components. Apicobasal polarity complexes are crucial for TJ establishment and maintenance; thus, there is an interplay between apicobasal polarity complexes and TJs. In intestinal epithelial cells, Spef1/CLAMP is present at the apical surface, cell–cell contacts, and the basolateral membrane. This pattern of localization depends on the degree of cell polarization. In sparse, migrating intestinal epithelia, Spef1/CLAMP is enriched at the basal membrane in stress fibers colocalizing with actin and focal adhesion sites. When epithelia reach full polarization and cell migration is reduced, Spef1/CLAMP is enriched at intercellular contacts and apical membrane domains (Figure 1E). At cell–cell contacts, Spef1/CLAMP colocalizes with and binds to adherens junction proteins, including E‐cadherin and p‐120 catenin, and to the TJ proteins ZO‐1, occludin, and cingulin. Spef1/CLAMP is enriched at the brush border of differentiated intestinal epithelial cells. It has been demonstrated that cytoskeletal proteins associated with apical junctional complexes regulate intestinal integrity and function.^41^, ^42^, ^43^, ^44^, ^45^ Therefore, the movement of Spef1/CLAMP to different membrane domains during the process of epithelial polarization suggests that it is involved in adhesion complex dynamics crucial to cell polarization, migration, and barrier function.^10^

Spef1/CLAMP regulates PCP signaling via asymmetric localization of cell polarity components (Figure 1C). In multiciliated morphant cells of Spef1/CLAMP, the asymmetric accumulation of PCP membrane proteins Pk2 and Dvl1 is lost.^12^ Interestingly, Spef1/CLAMP localizes symmetrically around the cell cortex and its depletion causes a bidirectional loss of Spef1/CLAMP and proper PCP signaling in neighboring cells, suggesting that Spef1/CLAMP modulates cell–cell interactions that facilitate the PCP pathway. Furthermore, Spef1/CLAMP leads to a significant loss of Celsr2, a cadherin which forms a complex with PCP members.^12^ Depletion of Celsr2 has no effect on Spef1/CLAMP localization, suggesting that Spef1/CLAMP function is upstream of PCP signaling. Spef1/CLAMP also contributes to the asymmetric distribution of MTs. In *Xenopus*, MTs form a dense apical network connecting dozens of basal bodies in multiciliated cells.^9^ This network comes into close proximity to the cell membrane at the posterior cell border. Spef1/CLAMP binds MTs, which is known to regulate upstream and downstream PCP signaling.^46^, ^47^ In tracheal and ependymal cells, treatment with nocodazole leads to a loss of PCP protein asymmetry.^47^, ^48^ *Xenopus* embryos treated with nocodazole do not exhibit changes in Celsr2 accumulation at the cell membrane. However, in Spef1/CLAMP morphant cells, the posterior enrichment of MT is lost and in the majority of these cells, the MT network organizes uniformly around the cell membrane, suggesting that Spef1/CLAMP regulates MT polarity asymmetry.^12^ These findings indicate that Spef1/CLAMP promotes the asymmetric distribution of both membrane‐associated PCP proteins and a stable subset of the MT network that is asymmetrically positioned within the cytoplasm.^12^

Cell migration is necessary for development and repairing cell injury in adulthood. Cells can migrate independently or in groups in a process known as collective cell migration. During collective migration, cells maintain cell–cell contacts, exhibit both morphological and behavioral polarization, and interact with neighboring cells. This process is important for morphogenesis and wound healing in adults. Cells from the skin in *Xenopus* migrate collectively toward the outer epithelia reaching the apical junctions. Werner *et al*. demonstrated that the Par complex (Par3/Par6/aPKC/Cdc42), essential for establishment of apicobasal cell polarity, is positioned at the leading edge of migrating cells and is required for apical positioning of centrioles during the intercalation process.^9^ Cells expressing a truncated version of Par3 (DN‐Par3) or ectopic expression of a kinase‐dead version of aPKC (aPKC‐KD) exhibit defects in centriole position and the direct movement of multiciliated cells into the outer epithelium.^9^ Importantly, Par‐defective cells exhibit a substantial loss of stable acetylated MTs at the apical area, suggesting that MTs accumulate at the apical side of intercalating cells in a Par‐dependent manner and that MT stability is critical during intercalation. Interestingly, Spef1/CLAMP localizes to the apical surface, where it stabilizes MTs of migrating multiciliated cells (Figure 1D). At the leading edge, Spef1/CLAMP colocalizes with components of the Par polarity complex. Cells expressing DN‐Par3 or aPKC‐KD have a substantial loss of Spef1/CLAMP at the apical surface. Depletion of Spef1/CLAMP decreases MTs, similar to Par‐defective cells. Importantly, Spef1/CLAMP morphant cells exhibit intercalation defects and reduced fluid flow on the surface of embryos due to reduction in the number of multiciliated cells.^9^ Thus, Par proteins mediate the apical localization of Spef1/CLAMP, which, in turn, leads to an accumulation of stable MTs at the leading edge that is required for the intercalation process of multiciliated cells in *Xenopus*.

Endogenous Spef1/CLAMP is enriched at the cell edge, lamellipodia, and filopodia of migrating intestinal epithelial cells (Figure 1F). In Spef1/CLAMP knockdown cells, actin appears as single disorganized filaments rather than as bundles at the leading edge.^10^ Depletion of Spef1/CLAMP also impacts the ability of cells to form lamellipodia. Downregulation of Spef1/CLAMP significantly reduces the number of filopodia at the leading edge and filopodia fail to maintain a forward direction, demonstrating random directionality and little to no tensile strength.^10^ These studies suggest that Spef1/CLAMP is crucial for filopodia and lamellipodia assembly, as well as the organization of actin filaments at the leading edge of migrating cells. These data are also consistent with Spef1/CLAMP being required for the reorganization of actin bundles in response to cell injury.

## CONCLUSIONS AND PERSPECTIVES

The functions of Spef1/CLAMP have been explored in several models (Figure 1) and it appears that, depending on its localization, this protein plays a crucial role in regulating cell movement. For example, Spef1/CLAMP is essential for flagellar and ciliary motility. MT‐binding and ‐bundling activities of Spef1/CLAMP are required for the formation of the CP apparatus and rotatory cilia. In association with MTs, polarity proteins, and actin cytoskeleton components, Spef1/CLAMP has been demonstrated to be an important mediator in cell migration, PCP signaling, and reorganization of the actin network. All these data suggest that Spef1/CLAMP is broadly involved in facilitating cell migration and multiple forms of cell polarity.

Although the mechanisms by which Spef1/CLAMP regulates all these functions within cells are unknown, we speculate that the functions of Spef1/CLAMP are largely dependent on its binding partners. Spef1/CLAMP contributes to the regulation of MT stability, and these data provide molecular insights into the mechanisms of this process. For example, the tip‐specific enrichment of Spef1/CLAMP in cilia suggests that this protein may exhibit plus‐end binding in growing cilia and through unknown factors may bundle the CP MTs to keep the MTs as a pair, thus facilitating CP MT elongation and preventing disassembly during cilia growth. The integrity of the CP apparatus is important for ciliary retention of CP proteins that may contribute to coordinated polarized rotatory movement of cilia.

Spef1/CLAMP also binds to actin and cytoskeletal proteins. However, we do not yet understand how Spef1/CLAMP assembles into actin and MT dynamic networks, nor have regulatory domains or factors that alter its affinity for these protein–protein interactions been identified. Although the functions of Spef1/CLAMP are not yet clear, we speculate that Spef1/CLAMP binds to MTs and moves along actin stress fibers, guiding the MT tips toward the cell edge during migration. Spef1/CLAMP may also function as a tether protein that could link cytoskeletal complexes to the leading edge. There also remains the question of whether the CH domain of Spef1/CLAMP mediates actin binding in a similar manner to how it regulates MT‐binding or whether there is a contribution of the CC domain, which is crucial for MT bundling and dimerization of Spef1/CLAMP. These questions open many exciting avenues for research regarding MT‐actin‐binding proteins and related cytoskeletal adaptor proteins.

## COMPETING INTERESTS

The authors declare no competing interests.

### AUTHOR CONTRIBUTIONS

R.T. and G.A.H. conceived the study concept and contributed equally to the critical revision of the manuscript.

### PEER REVIEW

The peer review history for this article is available at: https://publons.com/publon/10.1111/nyas.14845.
